# Anxiety Symptoms Predict Subsequent Depressive Symptoms in Neurodivergent Youth: A 10-Year Longitudinal Study

**DOI:** 10.1007/s10802-025-01292-3

**Published:** 2025-01-24

**Authors:** Stian Orm, Jeffrey J. Wood, Ingrid Nesdal Fossum, Keenan Adams, Per Normann Andersen, Krister Fjermestad, Merete Glenne Øie, Erik Winther Skogli

**Affiliations:** 1 Division Mental Health Care, Innlandet Hospital Trust, Brumunddal, Norway; 2 Department of Psychology, University of Inland Norway, Vormstuguvegen 2, Lillehammer, 2624 Norway; 3 Department of Education, University of California, Los Angeles, CA USA; 4 Department of Psychology, University of Oslo, Oslo, Norway; 5 Research Department, Innlandet Hospital Trust, Brumunddal, Norway

**Keywords:** Attention-deficit/hyperactivity Disorder, Autism, Depressive Symptoms, Anxiety Symptoms, Longitudinal

## Abstract

Neurodivergent youth often experience anxiety and depressive symptoms that may hamper adaptive functioning and well-being. There is little knowledge of how anxiety and depression are related in neurodivergent youth. Therefore, we aimed to examine whether the relationship between anxiety and depressive symptoms is uni- or bidirectional in neurodiverse youth. We assessed self-reported anxiety and depressive symptoms over time in 173 youth (*M*_*baseline age*_ = 11.7 years, *SD* = 2.1, 64% males, 36% females). The sample comprised 38 autistic youth, 85 youth diagnosed with attention-deficit/hyperactivity disorder (ADHD), and 50 comparison youth assessed at baseline (T1), 2-year follow-up (T2, 97% retention), and 10-year follow-up (T3, 73% retention). We used cross-lagged models to analyze the data. In neurodivergent youth, more anxiety symptoms at T1 and T2 predicted more depressive symptoms at T2 and T3. Preceding anxiety symptoms were linked with later depressive symptoms, even after accounting for autoregressive effects of depressive symptoms. The results are consistent with a prodromal model in which anxiety symptoms can independently foreshadow the emergence of depressive symptoms over the course of development among neurodivergent youth. Potentially, addressing anxiety symptoms among youth with autism or ADHD could play a role in preventing the onset of youth depression.

Autism and attention-deficit/ hyperactivity disorder (ADHD) are related neurodevelopmental phenomena (Hartman et al., 2016). Autism and ADHD are both associated with heightened anxiety and depressive symptoms across the lifespan (e.g., Andersen et al., 2015; Orm et al., 2021; Zendarski et al., 2021). A recent meta-analysis found a pooled prevalence of 35% for anxiety disorders and 19% for depression among individuals on the autism spectrum (Micai et al., 2023). A recent population study of 4743 youth with ADHD reported prevalences of 39% for anxiety disorders and 19% for depression (Danielson et al., 2024). Anxiety and depressive symptoms in youth on the autism spectrum and those diagnosed with ADHD (collectively referred to as neurodivergent youth in this paper) are associated with lower quality of life and poorer everyday functioning (Andersen et al., 2023; Lawson et al., 2020; Orm et al., 2023; Schei et al., 2016). Thus, knowledge about the developmental associations between anxiety and depressive symptoms could have important implications for clinical work and intervention efforts to improve mental health and well-being among neurodivergent youth (e.g., Wood & Gadow, 2010).

Anxiety and depressive symptoms are highly associated and often co-occur (Kalin, 2020; Saha et al., 2021). Conceptually, anxiety and depressive symptoms are both considered internalizing problems: upsetting thoughts and feelings experienced internally but not necessarily expressed through distressed behavior (Achenbach, 1966). Despite high co-occurrence and conceptual similarities, anxiety and depression are distinct categories of mental health difficulties (American Psychiatric Association, 2013). According to the tripartite model of anxiety and depression, the experience of negative affect is common to both, whereas they can be differentiated by the presence of physiological hyperarousal in anxiety and low positive affect in depression (Anderson & Hope, 2008; Clark & Watson, 1991).

Two competing perspectives on the relationship between anxiety and depressive symptoms in the general population have been articulated (see Jacobson & Newman, 2017 for review). First, the prodromal perspective postulates that maladaptive anxiety precedes and increases the risk of later depression (Hettema et al., 2006; Horn & Wuyek, 2010; Schleider et al., 2014; Starr & Davila, 2012). Second, the bidirectional perspective holds that anxiety and depression influence each other mutually. According to this second perspective, depression can precede and increase the risk of anxiety and anxiety can precede and increase the risk of depression (Kessler et al., 2008; Saha et al., 2021). In support of the bidirectional perspective, a meta-analysis of prospective studies found that anxiety symptoms predicted later depressive symptoms and that depressive symptoms predicted later anxiety symptoms (Jacobson & Newman, 2017). However, anxiety symptoms were a significantly stronger predictor of later depressive symptoms than vice versa. A limitation of the meta-analysis was that the authors were not able to control for the rank-order stability of symptoms over time (i.e., autoregressive effects) or concurrent associations between symptoms at baseline and follow-up.

A lens partly compatible with each of these perspectives holds that anxiety and depression are interrelated manifestations of the personality trait neuroticism (low emotional stability; Weinstock & Whisman, 2006), and would be expected to be intercorrelated, but to manifest at differing degrees depending on experience (e.g., experiences that trigger anxiety such as exposure to threatening circumstances or experiences that trigger depression such as loss, poor self-appraisal and lack of agency). However, because depressive disorders tend to emerge later than anxiety disorders in youth (Moffitt et al., 2007), perhaps reflecting increasing experiences that can trigger depression, early manifestations of anxiety could be an indicator of risk for subsequent depression even in the absence of childhood depression.

Studies that have controlled for autoregressive effects have reported mixed results. Choi et al. (2021) and Shi et al. (2022) found support for a bidirectional relationship between anxiety and depressive symptoms in neurotypical populations: adolescents (mean age 15 years) prospectively followed over 18 months and college students (mean age 18 years) prospectively followed over three months, respectively. In contrast, support for a unidirectional relationship between self-reported anxiety and subsequent depressive symptoms was found in a sample of neurotypical youth prospectively followed from 11 to 17 years of age (Lee & Vaillancourt, 2020). Differences in age groups and follow-up periods may explain the diverging findings. Studies with shorter follow-up periods seem to report a stronger bidirectional relationship than studies with longer follow-up periods, which seem to report greater support for the prodromal perspective. Thus, it may be that anxiety and depressive symptoms are more strongly co-influential over shorter than longer time spans. Furthermore, anxiety disorders are typically childhood-onset disorders, whereas depression more commonly emerges during adolescence and emerging adulthood (Kessler et al., 2005). Thus, from a developmental perspective, it seems likely that impairing anxiety in childhood more often precedes depression in adolescence or emerging adulthood.

Theoretically, it has been proposed that maladaptive anxiety could contribute to the development of depression through associated avoidance behaviors (Carvalho & Hopko, 2011; Jacobson & Newman, 2014; Trew, 2011). Anxiety promotes avoidance, which again prevents engagement in social activities and other community experiences (e.g., school, sport, hobbies, all of which can have embedded threat elements). Thus, anxiety and avoidance interfere with positive self-appraisals and mastery experiences. Without these, the cognitive elements of depression, such as self-doubt, are primed (Carvalho & Hopko, 2011; Trew, 2011). In support of this theoretical account, Jacobson and Newman (2014) found that anxiety predicted depression 12–14 years later among neurotypical individuals and that the relationship was mediated by avoidance behavior.

In the context of neurodivergent youth, little research has specifically examined the longitudinal relationship between anxiety and depressive symptoms. Among youth with ADHD, some evidence has suggested that anxiety symptoms attenuate the relationship between ADHD and depressive symptoms (Thapar et al., 2023). Such attenuation may indicate that anxiety symptoms precede and drive the emergence of depressive symptoms among youth with ADHD. However, to our knowledge, only one previous study has specifically examined the developmental relationship between anxiety and depressive symptoms in neurodivergent individuals. In a cross-lagged panel model, caregiver-reported anxiety symptoms in emerging adults with autism or developmental delay (mean age 20 years) predicted later depressive symptoms at a mean age of 26 years but not vice versa (Schiltz et al., 2023). This finding suggests that anxiety symptoms can be prodromes for later depressive symptoms. However, in an exploratory analysis of self-reports from a subset of the same sample, Schiltz et al. (2023) found evidence of the opposite; depressive symptoms in emerging adulthood predicted subsequent anxiety symptoms. Thus, the literature on both neurotypical and neurodivergent youth is mixed, and more research is needed on whether the prodromal or bidirectional models are empirically supported among neurodivergent youth. There is especially a need for studies using self-reported symptoms, as self-reports were only available for a subset of the Schiltz et al. (2023) sample and provided diverging findings to caregiver-reports.

## The Current Study

As noted by Schiltz et al. (2023), there is currently a critical gap in the literature regarding the temporal association between anxiety and depressive symptoms among neurodivergent individuals. Given the divergent findings of Schiltz et al. (2023) between self- and caregiver-reports, more studies are needed to reconcile the nature of this relationship in neurodivergent individuals. More studies are needed especially among youth, since anxiety and depressive symptoms may peak during adolescence and/or emerging adulthood (Schubert et al., 2017; Steinsbekk et al., 2022). As such, late childhood and adolescence may be a key developmental phase for preventive and treatment efforts (Creswell et al., 2020). Thus, the current study aimed to fill this gap in the literature by examining the developmental relationship between anxiety and depressive symptoms in neurodivergent and neurotypical youth. We used 10-year longitudinal data from the Lillehammer Neurodevelopmental Follow-Up study (LINEUP). We chose self-reports of anxiety and depressive symptoms because previous studies have suggested that parents may underreport anxiety and depressive symptoms compared to self-reports from neurodivergent youth (Andersen et al., 2015; Davidsson et al., 2017; Hurtig et al., 2009). We used structural cross-lagged regression models (Selig & Little, 2012) to simultaneously examine reciprocal, autoregressive, and concurrent effects of anxiety and depressive symptoms over time. Because of the divergent findings of Schiltz et al. (2023), we did not make any hypothesis about whether we would find a unidirectional or bidirectional relationship.

## Method

### Design and Procedure

The LINEUP study comprises three assessment waves, baseline (T1), 2-year follow-up (T2), and 10-year follow-up (T3). At each assessment, participants completed measures including self-reports of anxiety and depressive symptoms. Participants were recruited from child and adolescent psychiatric outpatient clinics at Innlandet Hospital Trust, Norway, upon consecutive referrals. All individuals aged between 8 and 17 years referred for assessment of autism or ADHD were invited to participate. The neurotypical comparison group was recruited through local schools and had to attend regular classes. All neurotypical youths had to screen negative for all present or lifetime psychological disorders in the semi-structured interview, the Kiddie-Schedule for Affective Disorders and Schizophrenia/Present and Lifetime version (Kiddie-SADS; Kaufman et al., 1997). Exclusion criteria for all participants were prematurity (< 36 weeks gestational age), having a disease affecting the central nervous system (e.g., epilepsy), or having IQ < 70. An additional exclusion criterion for the ADHD group was no history of stimulant treatment. This was because the baseline study focused on neuropsychological functioning and thus, a medication naïve sample was preferable for the study purpose of comparing the cognitive functioning of youth with ADHD to neurotypical youth prior to medication (Andersen et al., 2013; Skogli et al., 2014). Additional exclusion criteria for the neurotypical comparison group were dyslexia or head injury with loss of consciousness. The study was prospectively reviewed and approved by the Regional Committee for Medical Research Ethics in South-Eastern Norway (T1 and T2 ref. REK 6-2009-24; T3 ref. 2018/1611).

### Participants, Diagnostic Evaluation, and Treatment

At baseline, 38 autistic youth, 85 youth diagnosed with ADHD, and 50 neurotypical youth were included. Demographic and clinical characteristics of all three groups are presented in Table 1. Unfortunately, no data was collected on the participants’ ethnicity. There were no significant differences in age or sex between the groups, but the groups differed in IQ, mothers’ education, and ADHD and autism symptomatology. The diagnostic assessment at T1 was based on a semi-structured clinical interview (Kiddie-SADS; Kaufman et al., 1997) conducted separately with the youths and their parents, and supplemented with information from the Autism Spectrum Screening Questionnaire (ASSQ; Ehlers et al., 1999), the ADHD Rating Scale IV (ARS-IV; DuPaul et al., 1998a, b), the Child Behavior Checklist (CBCL; Achenbach & Rescorla, 2001), and teacher reports on academic and social functioning. All measures have demonstrated good psychometric properties in terms of diagnostic specificity and sensitivity (e.g., DuPaul et al., 1998; Kaufman et al., 1997; Nøvik, 1999; Posserud et al., 2009). Diagnostic assessment was conducted by experienced clinical psychologists and educational therapists. The assessments and diagnostic decisions were supervised and reviewed by a clinical neuropsychologist specialized in neurodevelopmental disorders. Disagreements were discussed to arrive at a ‘best estimate’ DSM-IV consensus diagnosis. Eight participants met the criteria for both ADHD and autism and were included in the autistic group. Among the participants with ADHD, six also met the criteria for an anxiety disorder and three for a depressive disorder. Among the autistic participants, 10 also met the criteria for an anxiety disorder and two for a depressive disorder.

**Table 1:** Demographic and clinical characteristics of the three groups at baseline, 2-year follow-up, and 10-year follow-up

T1 - Baseline	Autism *n* = 38		ADHD *n* = 85		NT *n* = 50		Group Comparison	
	*M*	*SD*	*M*	*SD*	*M*	*SD*	*p*	Post-hoc
Male/female (%)	32/6	(84/16)	46/39	(54/46)	32/18	(64/36)	0.006	
Age in years	12.0	2.3	11.6	2.1	11.6	2.0	0.532	
FSIQ	98.3	17.8	94.4	13.8	103.8	13.0	0.002	ADHD < NT
Mother’s year of education	12.8	2.7	12.7	2.2	14.6	2.4	< 0.001	Autism, ADHD < NT
ASSQ	21.5	9.2	9.7	10.1	1.6	1.9	< 0.001	NT < ADHD < Autism
ARS-IV	21.4	10.3	25.6	10.5	2.6	3.0	< 0.001	NT < Autism < ADHD

T2–2-Year Follow-up	Autism *n* = 37		ADHD *n* = 81		NT *n* = 50			
	*M*	*SD*	*M*	*SD*	*M*	*SD*	*p*	Post-hoc
Sample retention %	97.4		95.3		100.0			
Male/female (%)	31/6	(84/16)	43/38	(53/47)	32/18	(64/36)	0.006	
Age in years	14.2	2.4	13.6	2.1	13.6	2.0	0.342	
ASSQ	20.6	9.2	7.7	7.3	1.0	2.8	< 0.001	NT < ADHD < Autism
ARS-IV	14.4	8.7	18.2	10.9	2.2	2.7	< 0.001	NT < Autism < ADHD

T3–10-Year Follow-up	Autism *n* = 26		ADHD *n* = 61		NT *n* = 40			
	*M*	*SD*	*M*	*SD*	*M*	*SD*	*p*	Post-hoc
Sample retention %	68.4		71.8		80.0			
Male/female %	21/5	(81/19)	34/27	(56/44)	26/14	(65/35)	0.083	
Age in years	22.2	2.6	21.4	2.3	20.9	1.9	0.078	

*Note.* ADHD = attention-deficit/hyperactivity disorder; NT = neurotypical; FSIQ = Full Scale IQ, estimated from Wechsler Abbreviated Scale of Intelligence. ASSQ = Autism Spectrum Screening Questionnaire; ARS-IV = ADHD Rating Scale IV

Between the assessments, neurodivergent participants received treatment as usual (TAU) at the child and adolescent psychiatric outpatient clinic or in their community health and social services. Among the participants with ADHD, 44 participants (54%) received stimulant treatment at T2, and 7 participants (12%) received stimulant treatment at T3. Among autistic participants, 3 (8%) participants received stimulant treatment at T1, 4 participants (11%) received stimulant treatment at T2, and 2 participants (8%) received stimulant treatment at T3. At T1, none of the neurodivergent participants received antidepressants. At T2, one participant with ADHD (1%) and one autistic participant (3%) received antidepressants. At T3, six participants with ADHD (10%) and two autistic participants (8%) received antidepressants.

### Measures of Anxiety and Depressive Symptoms

At T1 and T2, we assessed anxiety symptoms with self-reports on the Revised Youth’s Manifest Anxiety Scale, second edition (Reynolds & Richmond, 1985). The RCMAS-2 comprises 49 items rated on a “yes” or “no” scale that can be summed up to a total anxiety score. At T3, we assessed anxiety symptoms with self-reports on the Adult Self-Report (ASR; Achenbach & Rescorla, 2003). The DSM-oriented anxiety subscale of the ASR comprises 9 items rated on a scale from 0 (absent), 1 (occurs sometimes), to 2 (occurs often). The change in measure from T2 to T3 was because data on the RCMAS-2, as a child-scale, was not collected at the 10-year follow-up, and thus was unavailable. The RCMAS-2 and the ASR have demonstrated good internal consistency (α ≥ 0.80 and α ~ 0.85, respectively) and concurrent validity in the form of strong correlations (*r* ≥.61) with other measures of anxiety in previous research on general population samples and samples of youth with anxiety (e.g., de Vries et al., 2020; Etkin et al., 2021; Guerrero et al., 2020). The RCMAS has also shown acceptable psychometric properties among neurodivergent youth specifically, in the form of good internal consistency (α = 0.88) and sensitivity to change following cognitive-behavioral treatment for co-occurring anxiety disorders (Chalfant et al., 2007; Mazefsky et al., 2011).

At all assessments, we assessed depressive symptoms with self-reports on the Short Mood and Feelings Questionnaire (SMFQ; Angold et al., 1995). The SMFQ comprises 13 items rated on a scale from 1 (not true) to 3 (true) and can be summed up to a total depressive symptoms score. The SMFQ has demonstrated good internal consistency (α = 0.87), a unifactorial structure, and good sensitivity (1.00) and specificity (0.79) among adolescents in a juvenile detention center and a unifactorial structure and good discrimination in an item response theory analysis among general population children (Kuo et al., 2005; Sharp et al., 2006). The SMFQ has also shown acceptable psychometric properties among neurodivergent youth specifically, in the form of good internal consistency (α = 0.85-0.87) and convergent validity with other measures of internalizing problems (Jarbin et al., 2020; Mazefsky et al., 2014).

### Data Analyses

We used analysis of variance (ANOVA) and chi-square test of independence to test for selective attrition based on baseline characteristics. To test the relationships between anxiety symptoms and depressive symptoms over time, we conducted multiple-group structural cross-lagged regression models (Selig & Little, 2012) in JASP version 0.19 (JASP Team, 2024). The models were estimated using robust maximum likelihood (MLR) to account for nonnormal distribution of data (Kline, 2023). The following effects were modeled: (1) Cross-lagged effects between anxiety and depressive symptoms at different times (e.g., anxiety at T1 as a predictor of depressive symptoms at T2 and depression at T1 as a predictor of anxiety symptoms at T2), while controlling for (2) autoregressive effects within each variable (e.g., depressive symptoms at T1 as predictor of depressive symptoms at T2), capturing the extent to which individual differences in symptoms are stable over time (i.e., rank order stability), and (3) concurrent associations between the variables at each assessment wave. Thus, the cross-lagged effects were the parameters of main interest. The cross-lagged pathways represented associations between anxiety symptoms and depressive symptoms from T1 to T2 and T2 to T3, allowing us to test both unidirectional and bidirectional relationships between the two variables. Lastly, due to selective attrition based on IQ, we included IQ as a covariate in the model by regressing IQ onto T1 anxiety and depressive symptoms.

We handled attrition by using full information maximum likelihood (FIML). Model fit was determined using the following indices: non-significant chi-square test of model fit, root mean square error approximation (RMSEA, should be ≤ 0.08), comparative fit index (CFI, should be ≥ 0.96), and standardized root mean square residual (SRMR, should be ≤ 0.09) (Alhija, 2010; Mueller & Hancock, 2001). We first tested a model where all effects (i.e., the cross-lagged and autoregressive effects and concurrent associations) were constrained across groups (i.e., neurodivergent youth and comparison youth). Thereafter, we tested whether the model fit improved when removing equality constraints of the cross-lagged effects and estimating them separately for neurodivergent and comparison youth. To interpret the effect size of the cross-lagged effects, we used the benchmark values established by Orth et al. (2024) with 0.03, 0.07, and 0.12 corresponding to a small, medium, and large effect, respectively. We expected large effect sizes, based on the effect sizes of 0.32 to 0.49 for the cross-lagged effects reported by Schiltz et al. (2023).We run a post-hoc power analysis using semPower (Moshagen & Bader, 2024). The power analysis (α = 0.05, *df* = 31, *p* = 24) indicated that we had adequate power (0.94) to detect misspecifications corresponding to a model with less than acceptable fit (RMSEA ≥ 0.08).

## Results

### Attrition

Of the 173 original participants, 168 participated at T2, giving an overall retention rate of 97.1% (see Table 2 for retention rates in each group). At T3, 127 of the original participants were re-assessed, giving an overall retention rate of 73.4%. Of the 46 original participants not re-assessed at T3, we were unable to locate 9 of them (4 from the ADHD group, 3 from the autistic group, 2 from the TD group) and 37 declined further participation (20 from the ADHD group, 9 from the autistic group, 8 from the TD group). We examined differences between those who participated at T3 and those dropping out in terms of age, sex, intellectual functioning, mothers’ educational level, and anxiety and depressive symptoms. Those who dropped out had significantly lower baseline IQ compared to those who were retained (*p* =.006).

**Table 2:** Baseline demographic and clinical characteristics between participants retained at 10-Year follow-up (T3) and participants not retained

	Retained *n* = 123		Non-retained *n* = 50		Group comparison
Baseline (T1)	*M*	*SD*	*M*	*SD*	*p*
Sex (% male/female)	59/41		53/47		0.674
Age in years	11.7	2.1	11.6	2.1	0.788
FSIQ	100.0	15.1	93.1	13.7	0.006
Mothers’ year of education	13.4	2.5	12.9	2.4	0.275
ADHD Rating Scale-IV	17.2	13.7	19.8	12.4	0.249
Autism Spectrum Screening Questionnaire	9.5	11.0	11.0	10.6	0.408
Revised Youth Manifest Anxiety Scale 2nd	13.2	9.2	13.0	9.5	0.870
Short Mood and Feelings Questionnaire	5.5	5.0	5.3	5.3	0.979

*Note.* FSIQ = Full Scale IQ estimated from Wechsler Abbreviated Scale of Intelligence

### Cross-Lagged Regression Models

See Table 3 for descriptive statistics on anxiety and depressive symptoms across the three assessment points. The first model with equality constraints for all effects exhibited unacceptable model fit (χ2 (35) = 41.82, *p* =.199, RMSEA = 0.09, CFI = 0.93, SRMR = 0.14). We modified the model by removing the equality constraints on the parameters of main interest, the cross-lagged effects, and let them be estimated separately for each group. The second model, where the cross-lagged paths were allowed to vary across group, showed acceptable model fit (χ2 (31) = 24.84, *p* =.775, RMSEA = 0.04, CFI = 0.99, SRMR = 0.10).

**Table 3:** Descriptive statistics for anxiety and depressive symptoms across the three groups and Assessment Waves

		Autism			ADHD			NT				
		*M*	*SD*		*M*	*SD*		*M*	*SD*		Group comparison	
											*p*	post-hoc
T1												
	Revised Youth’s Manifest Anxiety Scale 2nd	15.5	9.5		16.5	8.8		5.7	4.3		< 0.001	NT < Autism, ADHD
	Short Moods and Feelings Questionnaire	6.6	5.2		7.0	5.1		2.2	2.3		< 0.001	NT < Autism, ADHD
T2												
	Revised Youth’s Manifest Anxiety Scale 2nd	12.9	10.4		14.2	9.1		5.1	5.4		< 0.001	NT < Autism, ADHD
	Short Moods and Feelings Questionnaire	5.7	5.4		6.5	5.1		2.1	2.4		< 0.001	NT < Autism, ADHD
T3												
	Adult Self-Report, Anxiety Subscale	56.7	8.3		53.3	5.2		52.5	5.7		0.106	
	Short Moods and Feelings Questionnaire	8.5	7.3		7.7	6.8		3.4	3.6		< 0.001	NT < Autism, ADHD

Note. Note. T1 = baseline, T2 = 2-year follow-up, T3 = 10-year follow-up; ADHD = attention-deficit/hyperactivity disorder, NT = neurotypical

**Fig. 1:**
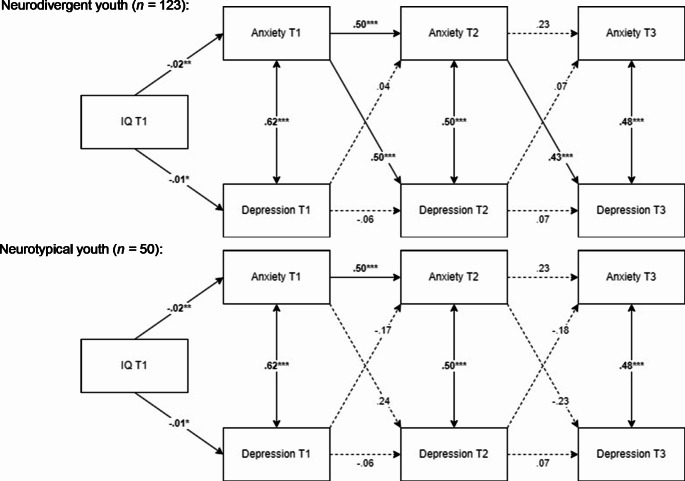
Results of structural cross-lagged regression analyses of the relationship between anxiety and depressive symptoms Note. ***p ≤.001, **p ≤.01, *p ≤.05. T1 = baseline, T2 = 2-year follow-up, T3 = 10-year follow-up. Solid lines indicate significant relationships, whereas dotted lines indicate non-significant relationships. All estimates are standardized

Figure 1 illustrates the final models. Among neurodivergent youth, anxiety symptoms at T1 (β = 0.50, *SE* = 0.10, *Z* = 4.89, *p* <.001, 95% CI [0.30, 0.71]) and T2 (β = 0.43, *SE* = 0.12, *Z* = 3.54, *p* <.001, 95% CI [0.19, 0.66]) significantly predicted subsequent depressive symptoms at T2 and T3, respectively, with large effect sizes. These results are consistent with the prodromal perspective which suggests that impairing anxiety may precede the onset of greater depressive symptoms over time in children and adolescents. Among neurotypical youth, all cross-lagged effects were non-significant. Across groups, there were significant and strong concurrent associations between anxiety and depressive symptoms at all assessment waves (*r* =.48 to 0.62, *p* <.001). From T1 to T2, a significant autoregressive effect was found for anxiety symptoms (β = 0.50, *SE* = 0.09, *Z* = 5.47, *p* <.001, 95% CI [0.32, 0.67]). However, when the time gap increased between T2 and T3, the autoregressive effect for anxiety symptoms was no longer significant (β = 0.23, *SE* = 0.12, *Z* = 1.83, *p* =.067, 95% CI [-0.02, 0.47]). This suggests, not surprisingly, that the rank-order stability in anxiety symptoms is stronger over short (i.e., two years) than longer (i.e., eight years) time spans.

**Fig. 2:**
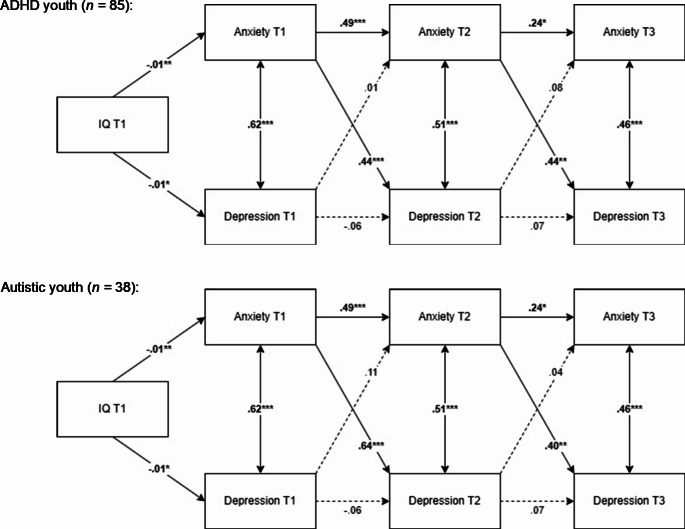
Results of structural cross-lagged regression analyses of the relationship between anxiety and depressive symptoms with separate estimates of the cross-lagged effects for autistic and ADHD youth Note. ***p ≤.001, **p ≤.01, *p ≤.05. T1 = baseline, T2 = 2-year follow-up, T3 = 10-year follow-up. Solid lines indicate significant relationships, whereas dotted lines indicate non-significant relationships. All estimates are standardized

As a supplementary analysis, we examined whether the results held up when dividing the neurodivergent youth into two separate groups, autistic youth and youth with ADHD. The results showed that the cross-lagged effects from anxiety symptoms to subsequent depressive symptoms were present in both groups (see Fig. 2). Thus, the main findings held up when the two groups were examined separately.

## Discussion

Our findings are consistent with a prodromal model of the relationship between anxiety and depressive symptoms in neurodivergent youth. More self-reported anxiety symptoms at baseline and the 2-year follow-up predicted more depressive symptoms 2- and 8-years later, respectively, after accounting for the stability of depressive symptoms over time (i.e., autoregressive effects). The effect sizes of the cross-lagged paths from anxiety symptoms to subsequent depressive symptoms were very large, whereas the effect sizes of the opposite cross-lagged paths from depressive symptoms to subsequent anxiety symptoms were in the small to medium range and non-significant. Thus, our findings suggest that, among neurodivergent youth, anxiety symptoms is a more important predictor of subsequent depressive symptoms than vise versa.

Our findings of a unidirectional relationship between anxiety symptoms and subsequent depressive symptoms in neurodivergent youth converge with the primary findings of Schiltz et al. (2023) among neurodivergent adults. However, Schiltz et al. found the opposite pattern in the subsample with available self-reports. Thus, our findings, using self-reports, diverge from their self-report findings. This could be due to a different age span examined in the current study. As noted in the introduction, depressive disorder onset tends to be in adolescence and early adulthood, so differing developmental processes may have accounted for their findings. Furthermore, as Schiltz et al. pointed out in their discussion, their subsample with available self-reports was necessarily different from their full sample.

The unidirectional relationship between anxiety and depressive symptoms among neurodivergent youth found in this study raises a question about *how* anxiety symptoms contribute to the development of or increase in depressive symptoms. As mentioned in the introduction, avoidance behaviors associated with anxiety play a key role (Jacobson & Newman, 2014). In line with behavioral theory, avoidance behaviors may prevent youth from experiencing environmentally-mediated social and non-social positive reinforcement, which subsequently can lead to depressive symptoms (Carvalho & Hopko, 2011).

Wood and Gadow (2010) proposed that core autism features such as social cognitive difficulties, repetitive behaviors, and sensory sensitivities can lead to aversive everyday situations, including punishing reactions and rejection from others, which subsequently leads to the development of anxiety and avoidance behaviors. This model can be extended to youth diagnosed with ADHD as they also experience social cognitive difficulties leading to peer rejection and sensory sensitivities, similar to many autistic youth (Lievore et al., 2023; Løkke, 2011; Panagiotidi et al., 2018). Furthermore, where the repetitive behaviors of autistic youth may contribute to critical or hostile reactions from others (Wood & Gadow, 2010), the same is true for the hyperactive and impulsive behaviors characteristic of ADHD (Glatz et al., 2011). Thus, a range of aversive experiences stemming from the core features of autism and ADHD may contribute to the development of anxiety and avoidance behaviors, which in turn reduces mastery experiences and positive reinforcement, and sets the stage for self-doubt and fewer experiences of positive affect– consistent with the depression-specific aspect of the tripartite model (Anderson & Hope, 2008). Further research is needed to address these hypotheses and examine more closely how anxiety symptoms contribute to the development of depressive symptoms among neurodivergent youth.

In the neurotypical comparison group, we found no significant cross-lagged effects between anxiety and depressive symptoms. A lack of significant findings in the neurotypical comparison group should be interpreted with caution since the neurotypical youths had to screen negative for all present or lifetime psychological disorders to be included in the sample. Thus, the lack of associations within this group may be due to floor effects. Furthermore, anxiety and depressive symptoms may be more fluctuating among neurotypical youth and this could make it difficult to detect temporal relationships over a longer time-span such as 2- and 8-years.

### Clinical Implications

The current findings have several clinical implications. First and foremost, the findings suggest that preventing and addressing anxiety symptoms in clinical work with neurodivergent youth may be indicated to avoid negative developmental cascades. Building on the model by Wood and Gadow (2010) and the neurodiversity framework (Sonuga-Barke, 2023), one approach could be the implementation of programs that aim to remove societal barriers for neurodivergent youth and increase acceptance of their differences. Such programs might prevent anxiety symptoms from developing by reducing the number of aversive everyday situations neurodivergent youth encounter. When anxiety symptoms emerge, anxiety treatment adapted to the needs of neurodivergent youth (e.g., Wood et al., 2020, 2021) may be important to prevent the development of depressive symptoms. A recent study of cognitive-behavioral treatment for youth with anxiety disorders found that the anxiety symptoms trajectory predicted the depressive symptoms trajectory, i.e., when anxiety symptoms increased or decreased, depressive symptoms developed accordingly (Fjermestad et al., 2024). Similarly, Wood et al. (2020) found that autism-adapted anxiety treatment was associated with less depressive and general internalizing symptoms compared to standardized anxiety treatment or treatment as usual. Thus, adequate treatment of anxiety in neurodivergent youth may lessen the development of depressive symptoms among those with underlying risk for internalizing difficulties.

### Limitations and Future Directions

The strengths of the current study include the controlled longitudinal design with a high retention rate 10 years after baseline assessment. Furthermore, the inclusion of autistic youth as well as those diagnosed with ADHD allows for the inclusion of a relatively large sample of youth with highly related manifestations of developmental diversity. Nonetheless, there are several limitations.

First, even though our sample size of neurodivergent youth was similar to that in the previous study by Schiltz et al. (2023; *N* = 130), the sample sizes of the individual groups are modest, and our findings should be considered preliminary. To preserve statistical power, we collapsed neurodivergent youth (i.e., autistic youth and youth with ADHD) into one group for the main analyses. This prevents us from saying anything about possible differences between the two groups of neurodivergent youth. As the study by Schiltz et al. (2023) also included several neurodevelopmental phenomena in the analyzed sample, there is still a question of whether different neurodevelopmental phenomena may differ in terms of the developmental relationship between anxiety and depressive symptoms. However, our supplementary analysis indicated that the main results held up separately in the autistic group and the group of youth with ADHD.

Second, although our sample size was similar to that of Schiltz et al. (2023), power may still be an issue. There are examples of cross-lagged regression analyses in the literature with samples as small as *N* = 54 (see Orth et al., 2024 for a recent review). However, the recommended sample sizes for structural regression models vary, but a common recommendation is *N* of ~ 200 (Baribeau et al., 2022). Our total sample size was just below the recommendation, and more considerably so if considering the neurodivergent and neurotypical youth as two separate samples. However, simpler models and larger effect sizes necessitate smaller samples than more complex models involving the estimation of latent variable parameters and subtle effects (Baribeau et al., 2022; Kline, 2023). In the current study, we used a relatively simple model comprising two manifest variables measured on three occasions, and we expected (and found) large effect sizes based on previous research (Schiltz et al., 2023). Thus, even though our findings should be considered preliminary, and replications are warranted, our findings contribute to a currently small part of the literature examining the developmental associations between anxiety and depressive symptoms among neurodivergent individuals.

Third, there was evidence of selective attrition due to lower IQ, which should be considered when interpreting our findings. At the 10-year follow-up, the sample comprised a subgroup that was relatively intellectually able. However, to control for this selective attrition, IQ was added as a covariate in the analyses. Fourth, our sample was clinically referred and may not be generalizable to the full population of neurodivergent youth. Thus, in conclusion, more research is needed to understand exactly how anxiety symptoms influence depressive symptoms in neurodivergent youth. A better understanding of this relationship may inform program development aimed at supporting the mental health and well-being of neurodivergent young people.

Fifth, our sample of youth with ADHD was stimulant naïve at baseline, and a stimulant naïve sample at the mean age of 11 years may differ from other clinical samples of youth with ADHD. For example, a recent systematic review suggested that a later age of diagnosis is associated with fewer co-occurring conditions (Rocco et al., 2021). Our sample also comprised more females (46%) than what is common in clinical samples (Faraone et al., 2015). This may be due to females being more likely to be diagnosed at a later age and less likely to be prescribed stimulants (Martin, 2024). However, a later diagnosis of females with ADHD may be partially due to diagnostic overshadowing, where females are more likely to be diagnosed with co-occurring anxiety and depression prior to receiving an ADHD diagnosis (see Martin, 2024 for a recent discussion). Also, although we lack data on the ethnicity of our participants, one should keep in mind that the sample was recruited from a rural part of Norway where the population is predominantly European-White. Thus, the particularities of our sample should be kept in mind when drawing conclusions and generalizations from our findings.

Lastly, we were limited by the available data. We were not able to examine possible explanatory mechanisms of the relationship between anxiety and depressive symptoms, such as avoidance, excessive worrying, and social aspects. Future studies should include a range of possible explanatory mechanisms to elucidate exactly how anxiety symptoms may contribute to subsequent depressive symptoms. Furthermore, in the future, it would be interesting to use the tripartite model of anxiety and depression (Anderson & Hope, 2008; Clark & Watson, 1991) and examine the temporal relationships between general negative affect, physiological hyperarousal, and lack of positive affect.
